# Counting multiple X-rays per pulse with an avalanche photodiode detector

**DOI:** 10.1107/S1600577525002462

**Published:** 2025-04-09

**Authors:** Liam T. Powers, Aidan M. Jacobsen, Stephen M. Durbin

**Affiliations:** a Department of Physics and Astronomy Purdue University West Lafayette IN47907 USA; b College of Electrical & Computer Engineering Purdue University West Lafayette IN47907 USA; Brazilian Synchrotron Light Laboratory, Brazil

**Keywords:** Avalanche photodiode detector, X-ray synchrotrons, digitized signal analysis

## Abstract

Avalanche photodiode detectors (APDs) at X-ray synchrotrons are typically limited to recording at most one photon per synchrotron pulse. Digitizing the APD amplifier outputs enables signal processing to accurately measure a mean count rate of at least four photons per pulse based on initial synchrotron measurements. Higher rates are readily achievable. This method allows APDs to be utilized for time-resolved measurements at much higher intensities than before.

## Abstract 2

Avalanche photodiode detectors at synchrotron sources can now be used at much higher count rates due to improved digital signal processing.

## 1. Introduction

Synchrotron sources produce X-rays in short pulses separated by significant delays. Pulse durations are generally around ∼10^2^ ps whereas the delay time between pulses depends on the synchrotron fill pattern and is typically between tens and hundreds of nanoseconds. Different timing structures provide opportunities for different types of time-resolved X-ray measurements, such as pump-induced changes in X-ray scattering, or separating prompt from delayed X-rays arising from a de-excitation process. Avalanche photodiode detectors (APDs) are especially well suited for many time-resolved studies because they can recover from detecting single X-ray photons before the next pulse arrives, allowing them to measure successive pulses as well as to distinguish between prompt and delayed X-rays (Kishimoto, 1992 ▸; Baron *et al.*, 2006 ▸). APDs are often described as having high count rate capabilities, but this actually refers to their ability to recover before the next pulse arrives; with few exceptions (Toellner *et al.*, 1994 ▸; Bordessoule & Lemonnier, 2002 ▸), they are typically constrained to measure at most one photon per pulse, with a maximum count rate thus limited by the pulse frequency (Kishimoto *et al.*, 2009 ▸; Li *et al.*, 2017 ▸). Instead of less than one photon per pulse, we report here on achieving mean count rates of about four photons per pulse and peak counts exceeding nine photons per pulse, with a technique than can be readily extended to higher rates.

The details of APD detection of X-rays have been well documented elsewhere (Baron *et al.*, 2006 ▸). In standard configurations, the absorption of a hard X-ray generates a current pulse that a fast, low-noise amplifier converts to a voltage pulse with a duration of several nanoseconds. Standard electronics can determine the X-ray arrival time with respect to a synchrotron timing signal. A discriminator is set to ensure a pulse height is in the range of a single X-ray photon, with the output read by a counter. Two photons arriving at the same time create a pulse too high to be accepted by the discriminator, suppressing the count rate. This pulse pile-up is carefully avoided by reducing the input X-ray intensity, keeping the output count rate proportional to the input signal.

Our scheme for accommodating pulse pile-up is conceptually simple and well understood: fast digitization of the amplifier output converts signal analysis from a hardware issue to a software issue. With a digitized output file, it is a simple matter to determine if a particular peak is from one photon or two, or if a peak was generated by a prompt or a delayed photon. Advantages of digitizing APD outputs have already been demonstrated by Kinigstein *et al.* for laser pump/X-ray probe studies (Kinigstein *et al.*, 2023 ▸; Kinigstein *et al.*, 2021 ▸). They measured laser-induced changes in X-ray absorption via detection of X-ray fluorescence and their digitization strategy allowed for greatly improved data collection efficiency.

## 2. Methods and results

**Figure 1:**
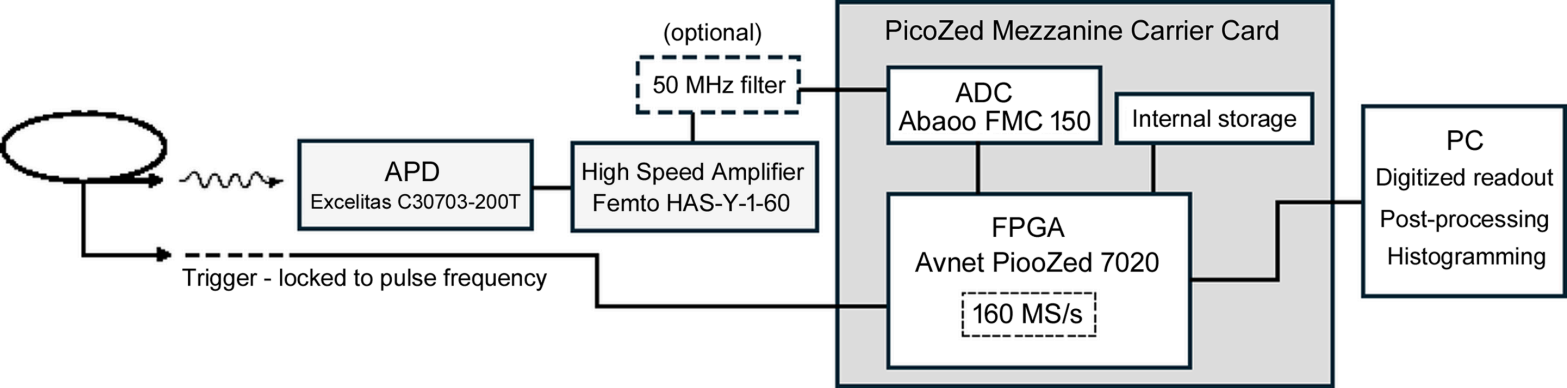
Detector system schematic. X-ray photons incident on an APD cause a current pulse that is converted by the amplifier into a fast voltage pulse. This is digitized by the ADC and written by the FPGA at 160 MS s^−1^ onto a time-ordered file on a memory card after a trigger pulse from the synchrotron control system. Datafiles can be exported and accessed on the PC via a network and processed for analysis. The optional low-pass 50 MHz filter can be inserted between the amplifier and the ADC to reduce high-frequency noise.

We address the challenge of accurately measuring the number of photons in each pulse when the mean number is greater than one. We remove the pulse pile-up limitation by processing the digitized amplifier output with a field programmable gate array (FPGA) for subsequent computer analysis. A conceptual schematic is shown in Fig. 1 ▸. The detection system consists of an APD device that converts an X-ray photon into a current pulse, a fast amplifier that produces a measurable voltage pulse, an analog-to-digital converter (ADC) to digitize the signal, and an FPGA system to control writing the data to a personal computer (PC) and other data manipulation.

Data were collected at the Advanced Photon Source in 48 bunch mode corresponding to ∼200 ps X-ray pulses separated by 77 ns intervals (13 MHz), with the APD simply exposed to the air scattering near an intense 8 keV X-ray beam. The APD (Excelitas C30703-200T) has an active area of 10 mm × 10 mm with a 200 µm thickness. Using published absorption data (Hubbell & Seltzer, 2004 ▸), this corresponds to an absorption probability and hence a quantum efficiency of 95.1% for 8 keV X-rays.

APD output pulses are amplified by a 1 GHz high-speed amplifier (FEMTO HSA-Y-1-60) to produce voltage pulses of 5–6 ns duration. This signal is sensed by an ADC (Abaco Systems FMC150) which was read at 6 ns intervals by the FPGA (Avnet PicoZed 7020 SOM) for an effective sampling rate of 160 MS s^−1^, well below the 800 MS s^−1^ capability of the ADC. The FPGA and ADC are connected via an Avnet PicoZed FPGA Mezzanine Connector Carrier card.

Though the FPGA can be configured to analyze each pulse and convert it to an integer equal to the number of photons in that pulse, it proved more effective to simply have the FPGA record the digitized data and conduct the signal analysis after recording. Additional APD inputs can be easily added to this setup, allowing for simultaneous multi-channel pulse-by-pulse analysis.

Data were collected in 500 ns segments triggered by a synchrotron timing signal. The total accumulation time for each dataset discussed below was 5.0 ms, limited in particular by the internal memory storage capacity of the FPGA. After being written to the PC hard drive, the data were analyzed by a computer program that simply creates a histogram of voltage levels for each data point (recorded every 6 ns, including the time between X-ray pulses). The histogram bin size was set to be much smaller than the pulse height of a single X-ray photon.

**Figure 2:**
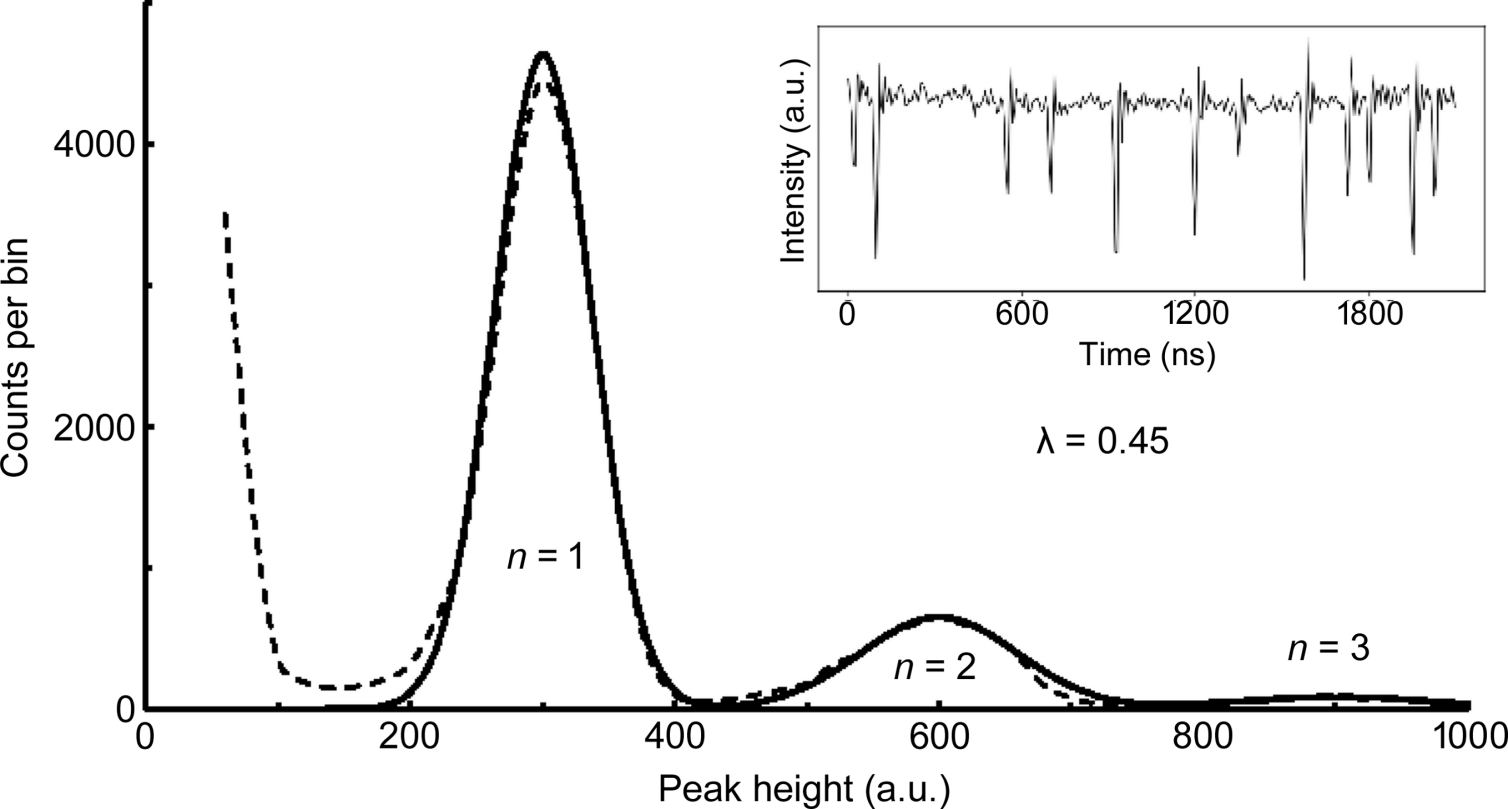
Pulse-height histogram of photons per synchrotron bunch. Dashed line – histogram of the digitized APD pulses showing the relative likelihood of 1, 2 and 3 photons per bunch arriving at the APD. The APD recorded air-scattered X-rays from an 8 keV synchrotron beam. Solid line – fit assuming a Poisson distribution with a mean value λ = 0.45 photons per bunch. Inset: digitized amplifier output for a 2 ms time window, recorded with a 50 MHz low pass filter to reduce baseline noise.

A dataset for one APD is given by the dashed line in Fig. 2 ▸, created by histogramming the raw data such as the subset shown in the inset. These data were obtained with a noise-reducing 50 MHz low-pass filter after the amplifier. A moderate count-rate was selected to keep multi-photon peaks clearly separated for more accurate analysis. These data were modeled by the sum of three Gaussians and it was found that their widths increased linearly as
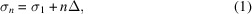
where σ_1_ is the standard deviation of the one-photon peak,*n* is the number of photons in a multi-photon peak and the constant Δ (for *n* > 1) was determined by a fit to these data. Signal baseline was determined from recordings with the beamline shutter closed, corresponding to zero voltage output. The centers of the Gaussians are equally spaced. We assume that the area under the *n*th Gaussian is proportional to the probability of an X-ray pulse having *n*X-ray photons. We also require that this probability is given by Poisson statistics, where the probability of *n* photons is given by 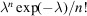 with λ being the mean number of photons per pulse. The Gaussian amplitudes are thus determined by a single fit parameter λ, which was λ = 0.45 photons for this dataset (corresponding to a 5.9 MHz count rate).

**Figure 3:**
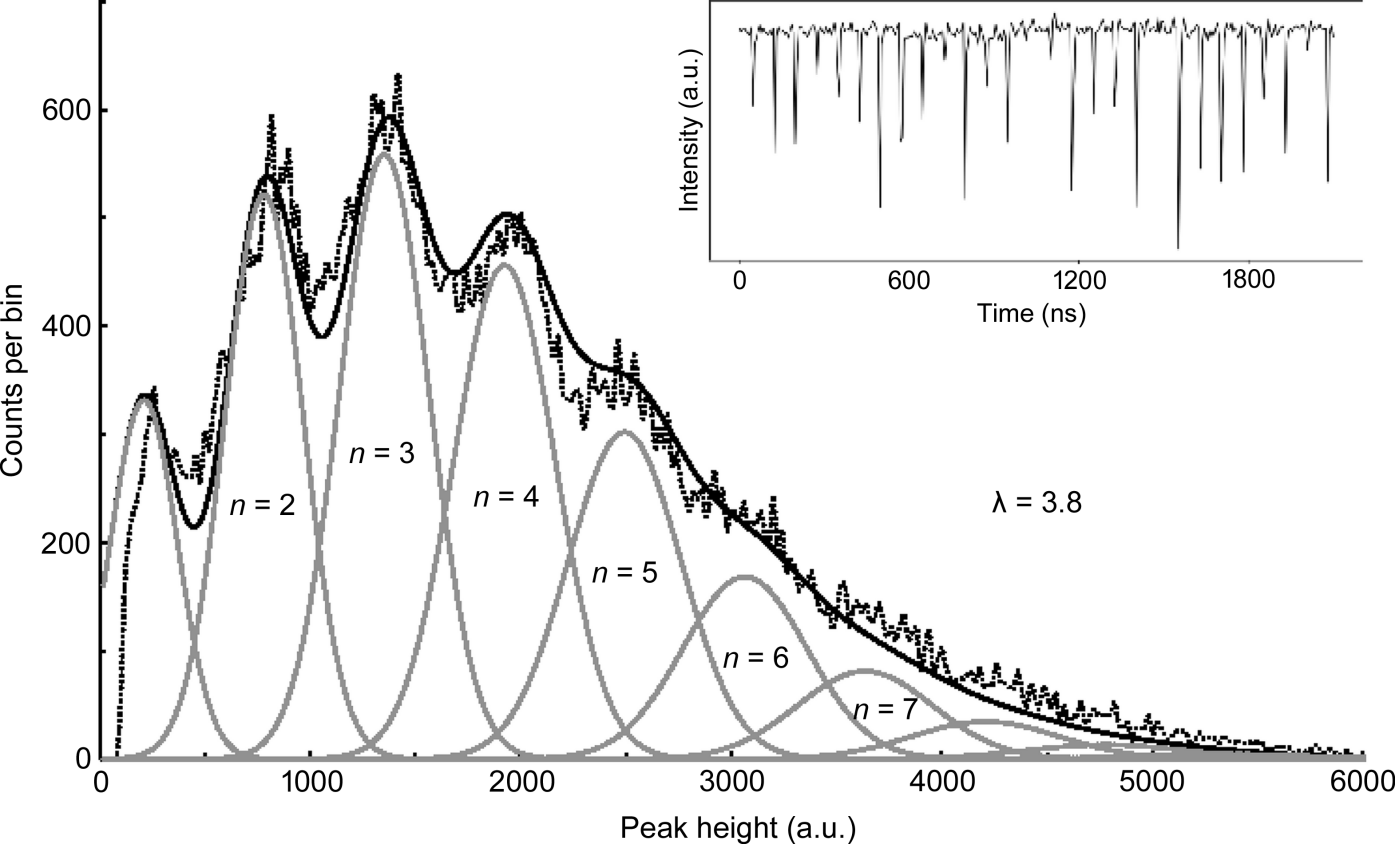
High-intensity pulse-height histogram. Dashed line is the histogram of digitized APD pulses recording a higher rate of air-scattered X-rays (achieved by moving the APD closer to the 8 keV synchrotron beam). These data are modeled by a series of peaks containing from one up to nine photons per bunch, equally spaced and with linearly increasing widths (see text). The relative amplitudes are fixed by assuming Poisson statistics. The solid line is the best fit to the data obtained by setting λ = 3.8. Inset: digitized amplifier output for a 2 ms time window.

A higher air scattering count-rate was obtained by positioning the APD closer to the X-ray beam path. Fig. 3 ▸ shows this higher count rate configuration, where the dashed line is the histogrammed data, with the inset again showing a slice of the raw data. These were acquired without a low-pass filter to reveal the full noise content of the signal. Only the first few peaks can be clearly identified because the broadening with increasing *n* increases the overlap with neighboring peaks. These data are again fit by a sum of Gaussians with widths governed by equation (1), centers equally spaced and amplitudes constrained by Poisson statistics. A best-fit analysis found the mean number of X-rays per pulse to be λ = 3.7 (corresponding to 48 MHz).

**Figure 4:**
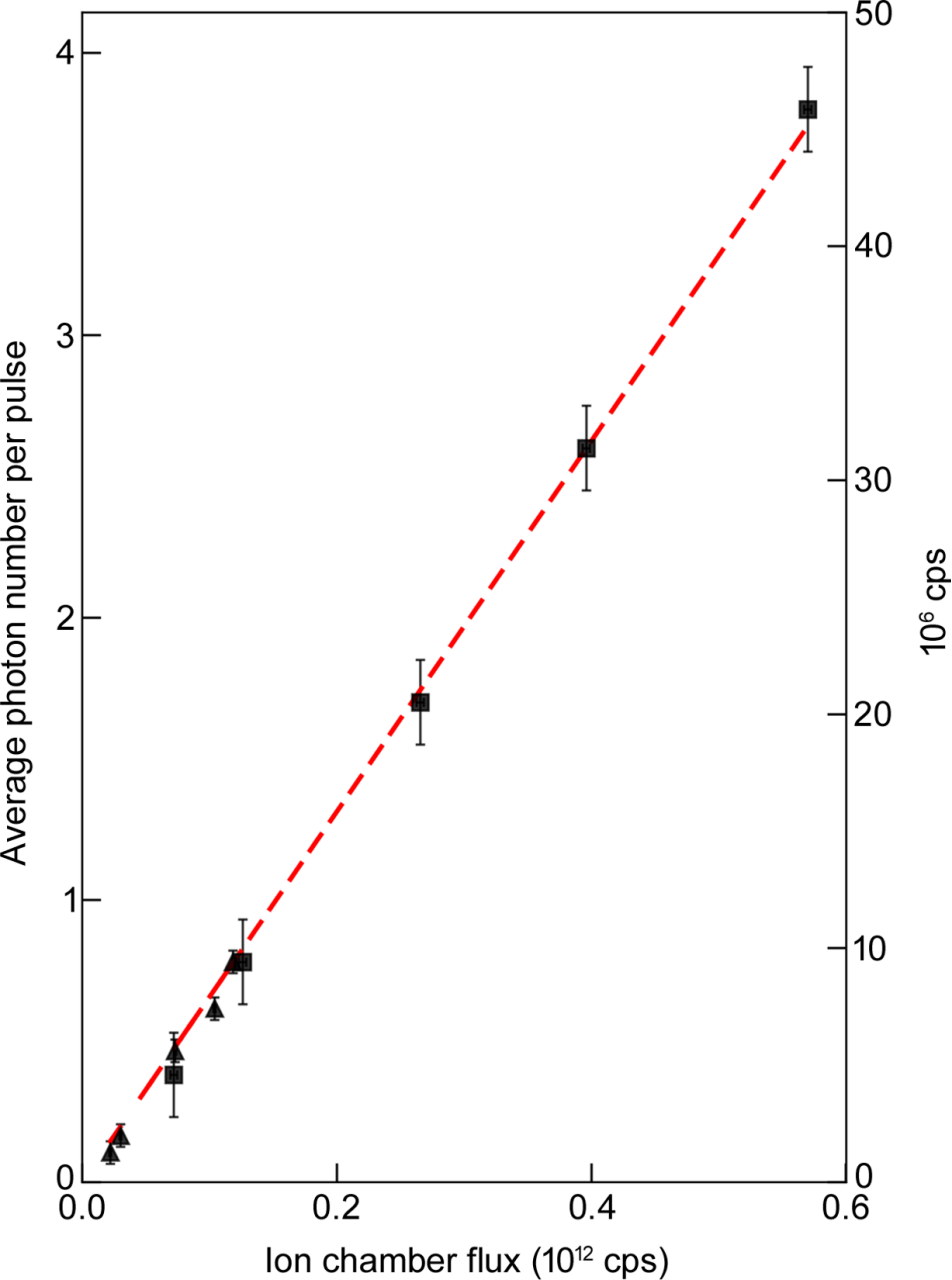
Linearity of λ versus detector input rate. The mean number of photons per pulse (λ) due to air scattered X-rays as determined by the Poisson fits (*e.g.* Figs. 2 ▸ and 3 ▸) are plotted against the direct beam flux generating the air scattering. Triangles denote data acquired with the detector positioned farther from the direct beam, whose intensity was adjusted by changing the beam-defining slits. Squares denote equivalent data with the detector positioned closer to the beam. The right axis gives the equivalent number of photons per second for the 13 MHz synchrotron bunch frequency. *y*-axis error bars show the range over which no change is observed in the best fit of the Poisson model to the data; *x*-axis error bars due to incident beam intensity fluctuations are smaller than the symbols. (The ion chamber output was corrected for a small offset, ensuring the linear fit goes through the origin.)

The intensity of the 8 keV beam creating the air-scattered X-rays was varied by changing the beam-defining slits in discrete steps, ranging from 0.05 to 0.60 × 10^12^X-ray photons per second as determined by a calibrated ion chamber. Values of λ from measurements at two different distances from the direct beam (compare Figs. 2 ▸ and 3 ▸) are plotted in Fig. 4 ▸ for this range of intensities. To determine the linearity of the APD response, we fit the λ values for the farther separation (weaker signals) versus the direct beam intensity; good linearity is observed from 0.1 to 0.7 photons per pulse, as expected. The second dataset (stronger signals) ranging from 0.4 to 3.7 was scaled to match the first dataset. These also show excellent linearity. There is no evidence for a non-linear response up to 4 photons per pulse. We also note that no measures were taken to regulate the temperature of the APD, which can be important for ensuring a linear response. Such measures may be necessary to extend the observed linearity to higher photon rates.

Finally, we note that the mean number of photons per pulse determined from the APD output is not quite the same as the mean number entering the APD if the detector efficiency is less than unity. When the incident photon flux has a Poisson distribution with mean value of λ, it can be shown that the detector output pulses will have a mean value of βλ, where β is the detector quantum efficiency. We assume that β, for the APDs used here, is the same as the probability of an 8 keV X-ray being absorbed by 200 µm of silicon, approximately 95.1%, so any losses would be negligible.

## 3. Conclusions

This APD signal-processing scheme combines digitizing the amplifier output with Poisson statistics applied to multi-photon peaks. We draw several conclusions from these results. Primarily, the problem of pulse pile-up has been removed even for pulses containing an average of four photons per pulse, including pulses with up to nine photons. A linear detector response is expected for even higher count-rates. A data acquisition time of 5 ms was sufficient to accurately determine the mean number of photons per pulse and hence the total count-rate. The data acquisition time could be extended to essentially continuous read-out and data processing with commercially available systems that have more internal memory, better integrated ADC functions, and more efficient protocols for reading and writing data at higher rates, such as the *Acqiris SA120P* system similar to that used by Kinigstein *et al.* (2021 ▸).

Thus, the maximum APD count rate is no longer constrained to be less than the synchrotron bunch frequency as long as the spacing between bunches is greater than the width of the voltage pulse produced by the amplifier electronics, about 6 ns for the results presented here. Significant effort by other groups has been put into improving the time resolution of APD detectors (Li *et al.*, 2017 ▸) which may enable this new approach to be possible for synchrotrons with bunch spacings less than 6 ns.

## Data Availability

All data are available upon reasonable request sent to the corresponding author.
